# Neurobiological effects of perceived stress are different between adolescents and middle-aged adults

**DOI:** 10.1007/s11682-020-00294-7

**Published:** 2020-08-01

**Authors:** Jingsong Wu, Horace Tong, Zhongwan Liu, Jing Tao, Lidian Chen, Chetwyn C. H. Chan, Tatia M. C. Lee

**Affiliations:** 1 College of Rehabilitation Medicine, Fujian University of Traditional Chinese Medicine, Fuzhou, China; 2 Brain Hospital, Guangzhou Medical University, Guangzhou, China; 3 State Key Laboratory of Brain and Cognitive Sciences, The University of Hong Kong, Hong Kong, China; 4 Laboratory of Neuropsychology and Human Neuroscience, The University of Hong Kong, Hong Kong, China; 5 Fujian University of Traditional Chinese Medicine, No. 1 Huatuo Road Shangjie Minhou, Fuzhou, 350122 China; 6 Applied Cognitive Neuroscience Laboratory, Department of Rehabilitation Sciences, The Hong Kong Polytechnic University, Hong Kong, China

**Keywords:** Perceived stress, Voxel-based morphometry, Plasma cortisol, Adolescents, Orbitofrontal cortex

## Abstract

Stress is an inevitable element of everyday living. Developmental studies suggested that adolescents are more vulnerable and sensitive to the effect of stress due to their developing brains, especially in areas related to stress perception and processing. This voxel-based morphometry study examined the association between various neurobiological markers and the level of perceived stress experienced by adolescents (*n* = 26) and middle-aged adults (n = 26). Our findings indicated that differences existed in the relationships between perceived stress and the structural volume of the orbitofrontal cortex (OFC) extending to the insula and amygdala. Specifically, the levels of perceived stress and the grey matter volume of the orbitofrontal cortex, the insula, and the amygdala were positively related in adolescents but negatively related for adults. Furthermore, a significant negative correlation between perceived stress and cortisol levels was observed in adults, whereas the relationship between perceived stress and cortisol levels was not significant for adolescents. Perceived stress measurement may be better than cortisol levels in terms of reflecting the emotional states of adolescents. In sum, the relationships between perceived stress and neurobiological markers were different between adolescents and middle-aged adults and thus appeared to be age dependent.

## Introduction

Stress is an inevitable life experience that leaves marks on the brain and hence shapes behaviors, leading to long-term impacts on mental health (Teicher et al. 2016). Stress response is typically triggered by the onset of a stressful event (i.e. the stressor), which induces the stress reactions via the individual’s appraisal of the stressor (Kalisch et al. 2015, 2017). For example, while two individuals may both have recently lost their jobs, their reactions to the situation would depend principally on their appraisal of the situation. The individual who perceives the job loss as being resulted from his/her own inadequate ability, faulty personality and thus has low confidence of finding a new job would experience stronger negative stress reactions than the individual who perceives the situation as being partly due to bad luck or an opportunity for personal growth. Cohen et al. (1983) proposed that stress occurs only when an individual perceives a situation to be threatening and no coping resources are available. They then developed the Perceived Stress Scale (PSS) to emphasize that it is the appraisal of an event rather than the event itself that brings about individual variation in stress responses.

Previous functional imaging studies identified the possible neural correlates of affective processing that perceived stress could affect. Maltreated children are associated with smaller orbitofrontal cortices (OFC) compared with non-maltreated children, suggesting that alterations in the OFC might lead to the maladaptation of an individual to dynamic social contexts (Hanson et al. 2010). A meta-analysis revealed that the OFC is one of the areas that differs the most when one compares volumetric differences between depressed individuals and healthy controls (Koolschijn et al. 2009). The ventromedial prefrontal cortex (vmPFC) is another area that is vulnerable to stress response. Admon et al. (2013) showed that military servicemen with posttraumatic stress disorder (PTSD) have reduced functional and structural connectivity between the hippocampus and the vmPFC. In addition, the results of a meta-analysis indicated that the structural volumes of the vmPFC as well as the anterior cingulate cortex (ACC) were smaller in people suffering from PTSD, relative to healthy controls (Kühn and Gallinat 2012). The insula cortex is an area associated with interoceptive awareness and subjective feelings (Craig 2009a). Ansell et al. (2012) investigated the effect of stress on the insula, and the results showed a negative association between recent stressful events and the insular volume. The morphometric results from Critchley et al.’s study (2004) showed that the grey matter volume in the right insula is linked to sensitivity to bodily responses (heartbeat rate) and subjective emotional experiences. The activity of the amygdala is implicated in threat perception. The hyperactivity of the amygdala is observed in patients with PTSD and social anxiety disorder (Etkin and Wager 2007). Based on the literature, structural volumes of regions including the OFC, vmPFC, ACC, insular, hippocampus, and amygdala could be sensitive to perceived stress.

Rao et al. (2010) reported relationships between decreased hippocampal volume and early-life adversities and proposed an interaction model among the size of the brain, particularly the hippocampus, childhood adversity, and vulnerability to depression. Furthermore, De Brito et al. (2013) observed that relative to healthy-age peers, adolescents experiencing childhood maltreatment showed overall smaller grey matter volumes, especially in the medial orbitofrontal cortex (mOFC) and the bilateral middle temporal gyrus (mTPG). Human’s brains undergo active neuroplastic changes throughout their lifespans. Therefore, the impact of stress on adolescent and adult brains could be very different. For example, adults who had experienced childhood maltreatment showed volume reduction in the hippocampus. However, this change was not reliably observed in children and adolescents suffering from maltreatment (Woon and Hedges 2008). Reductions in PFC volumes were consistently observed in adults who had experienced childhood maltreatment. However, findings on the PFC volumes in maltreated adolescents were very mixed. For example, De Bellis et al. (1999) showed no volume differences in the PFCs of maltreated versus non-maltreated adolescents. Carrion et al. (2009) observed more grey matter in the PFCs of maltreated adolescents. These inconsistent findings suggest that the effect of childhood maltreatment may not be apparent in some brain regions until adulthood, and the functionality of emotional processes in the subcortical networks and correlates, for example, the amygdala, may not be fully mature during adolescence (Passarotti et al. 2009). Based on the different degrees of neuroplasticity of adolescent and adult brains, the effect of perceived stress on these two age groups should be different.

Cortisol, among all hormones, is strongly associated with negative life contexts, such as stress. Smyth et al. (1998) found that an elevated salivary cortisol level was associated with the response to a stressful event, negative affect, and the anticipation of a stressful event in the future. Wahbeh and Oken (2013) showed that veterans with PTSD have lower salivary and urinary cortisol levels at all times compared with non-PTSD veterans. The recent literature showed that the relationship between cortisol and stress is a sophisticated one, which could be modulated by the maturity of the brain. Indeed, early stressful events could impair the later effects of hormones in adolescence, resulting in a weakened ability to cope with the social environment (Kolb et al. 2012). Halligan et al. (2007) showed that early adverse life events could influence morning cortisol secretion years later by altering the hypothalamic–pituitary–adrenal (HPA) activities in the early developmental stage. Pervanidou and Chrousos (2012) suggested that the development of the structure and function of the brain and cortisol are inter-linked. Because the emotional system in the brain (e.g., the PFC and the amygdala) of an adolescent is in maturation and is vulnerable to stress, it is more likely that the cortisol level will be dysregulated. The chronic abnormality of cortisol secretion would then affect the pubertal development of the brain and cause obesity.

From the literature reviewed above, we speculated that age-related differences would exist in the relationships between perceived stress and the structural volumes of the regions of interest (ROIs), including the OFC, vmPFC, insula, ACC, hippocampus, and amygdala, in adolescent and adult brains verified by this cross-sectional neuroimaging study using voxel-based morphometric analysis. Specifically, we speculated that adolescent brains are more vulnerable to stress and hence would show more significant volume changes in the ROIs of this study. In addition, we speculated that significant relationships would exist between cortisol levels and perceived stress observed in adults but not in adolescents.

## Materials and methods

### Participants

This study received approval from the Fujian Traditional Chinese Medicine University Institutional Review Board. Fifty-two healthy, right-handed Chinese participants recruited from the community via advertisement participated in this study. The inclusion criteria were as follows: (1) right-handed (using the Edinburgh Handedness inventory); (2) no history of neurological neurodegenerative or psychiatric diseases affecting neurocognitive and/or affective functioning; (3) normal or corrected-to-normal vision and hearing; (4) no history of substance abuse; and (5) physically and psychologically suitable for magnetic resonance imaging (MRI) scanning. All subjects provided written informed consent prior to participation. We have additionally obtained parent’s consent for those participants who were under the age of 18. Experimental procedures were conducted according to the Declaration of Helsinki. All participants arrived at the hospital in fasting state in the morning, and a nurse collected their blood sample. They then completed the Test of Nonverbal Intelligence, third edition (TONI-III), the Chinese version of the Perceived Stress Scale (PSS), and provided the experimenter with general demographic information. MRI scanning then followed as the last step of the data collection.

### Perceived stress

We used the Chinese version (Yang and Wang 2003) of the PSS (Cohen et al. 1983) to assess the subject’s perceived stress. The PSS has been proved to be suitable for adolescents, adults, and the elderly, and it focuses on assessing the respondent’s stress-related feelings, experiences, and emotions. The PSS contains 14 items on a five-point Likert scale ranging from never (0) to very often (4), with higher scores representing higher elevations of perceived stress. The total score ranges from 0 to 56, with a higher score corresponding to a higher degree of subjective stress. We assessed general perceived stress, and therefore, participants were asked how they “generally” felt about each item on the scale. Each participant completed the scale prior to MRI scanning. It showed good internal consistency in the current sample with Cronbach’s alpha = 0.81.

### Plasma cortisol levels

A registered nurse collected peripheral blood (5 ml) from each participant with EDTA tubes from 0800 to 0900 in the morning. Blood samples were then immediately centrifuged at 3000 rpm (rpm) for 10 min at room temperature to extract plasma. The plasma was then collected and stored at −80 °C for further analysis. All cortisol assays were performed using an enzyme-linked immunosorbent assay kit from Quansys Biosciences (Logan, UT, USA). The detection range of the kit was 1.06–775 ng/ml. The average intra-assay coefficient of variation (CV) was 6.3%, and the average inter-assay CV was 6.0%.

### MRI image acquisition

High-resolution T1-weighted anatomical images were acquired with a 3.0 T GE MRI scanner (GE Medical Systems, Erlangen, USA) with an eight-channel GE head coil. The magnetization-prepared rapid gradient echo (MPRAGE) sequence was used (TR/TE/TI = 2000 ms/1.75 ms/450 ms; flip angle = 15°; slice thickness = 1 mm; FOV = 240 × 240 × 160 mm; resolution matrix = 256 × 256; voxel size =1 × 1 × 1 mm). Subjects were instructed to keep still and awake for the entire duration of the scanning, which lasted 5 min.

### MRI image analyses

Voxel-wise analysis of cerebral grey volume was based on SPM12 (https://www.fil.ion.ucl.ac.uk/spm/software/spm12/) running on MATLAB (version 2015b) (Mathworks Inc., Natick, MA, USA) were conducted. Each MRI image was first screened for artifacts or anatomical abnormalities in SPM12. Structural images of each subject were then preprocessed according to the following steps: (1) brain tissue segmentation with an integrated approach, including bias correction, image registration to the MNI template, and tissue classification into grey matter, white matter, and cerebrospinal fluid; (2) brain skull-stripping with the light cleanup method; inter-subject registration using diffeomorphic anatomical registration through exponential lie algebra (DARTEL) with improved performance (Ashburner 2007); (3) “modulation” by scaling the warped images with the Jacobian determinants derived from the registration step; and (4) data smoothing with an 8-mm full width at the half maximum isotropic Gaussian kernel. The “modulation” step allows for the tissue volume to be preserved after warping. The smoothing step further reduces inter-subject misalignment and renders normally distributed images so that the assumption of parametric statistical comparisons is not violated. To calculate the Total intracranial volume (TIV), the total grey matter volume (GMV), white matter volume (WMV) and cerebrospinal Fluid (CSF) volume were obtained for each participant. TIV was calculated as the sum of GM, WM and CSF volumes.

The smoothed grey matter images were submitted to second-level general linear model (GLM) analysis using SPM12: v = Age + Gender + IQ + TIV + PSS + Age × PSS, where age includes two levels (adolescence vs. adults), gender, TIV and IQ are controlling covariates, and the PSS score is the covariate of interest. To account for Type I error, both FWE correction in SPM12 with uncorrected *p* < 0.001 and non-parametric permutation inference using “randomize” (uncorrected *p* < 0.001, number of iterations = 10,000) in the FMRIB Software Library (Smith et al. 2004) were conducted on a whole brain level on the preprocessed data.

To examine whether PSS scores are related to brain imaging properties, correlation analyses were performed between the measures of the PSS score and resulting imaging indices from VBM using SPSS 24.0 (IBM Corporation, Armonk, NY).

## Results

### Participants and behavioral measures

The final sample consisted of 26 adolescents ranging from 14 to 17 years old (13 females; *M*_*age*_ = 16.85 ± 0.21 years) and 26 adults ranging from 30 to 45 years old (13 females; *M*_*age*_ = 36.74 ± 5.23 years). These two groups were matched in terms of gender composition, PSS general (*p* = .11), and intellectual abilities measured by the Test of Nonverbal Intelligence, Third Edition (TONI-III) (*p* = .24) (Table 1).

**Table 1:** Demographic and psychometric characteristics of the participants

Characteristic	Adolescent (*n* = 26)	Adult (*n* = 26)	Statistical Analysis
Age (Years)	16.85 ± 0.21	36.74 ± 5.23	*t* = −18.99*
			*p* < 0.01
Gender (M/F)	13/13	13/13	*t* = 0
			*p* = 1.00
IQ (scores)	105.58 ± 14.12	109.00 ± 11.89	*t* = −0.946
			*p* = 0.24
PSS_A_ (scores)	26.38 ± 7.01	23.38 ± 8.84	*t* = 1.36
			*p* = 0.11
Cortisol_A_ (scores)	17.04 ± 7.07	13.09 ± 5.07	*p* = 0.12

IQ is measured by TONI-III; PSS, Perceived Stress Scale

Data are expressed as mean ± standard deviations

_A_ Gender and IQ were added as covariates in the analysis

*indicates a significant statistical difference with *p* < 0.05

### GMV and stress

**Fig. 1:**
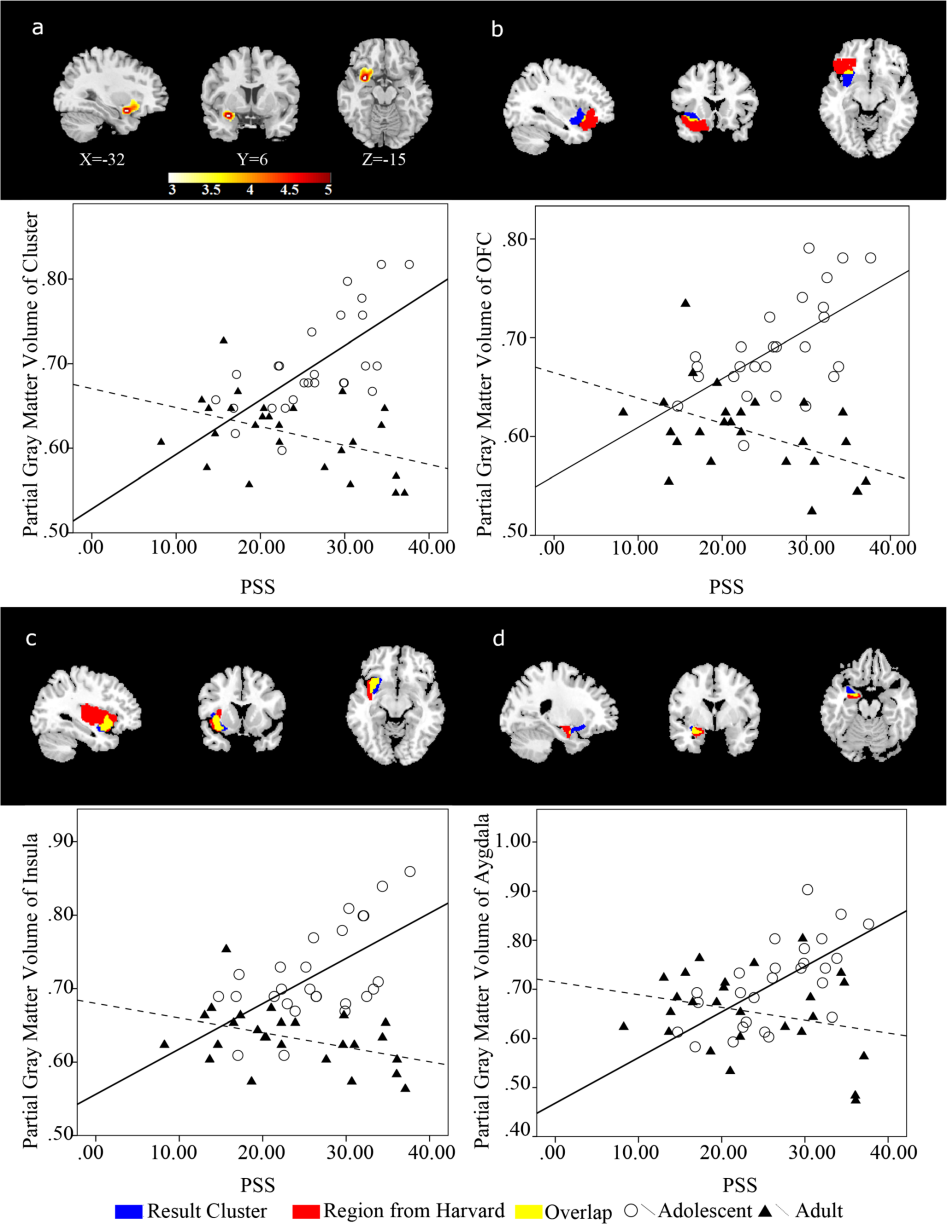
Results of MRI Image Analysis. a β-value map according to the interaction of PSS score and the age group to the grey matter volume in VBM analysis, and the partial residual plot showing the relationship of PSS score versus cluster grey matter volume in two age groups. The components attributed to covariates (age, gender and IQ score) are subtracted from volume value and only the one predicted by PSS score plus residual were kept. b The overlap of the cluster and Left OFC, and the partial residual plot between PSS score and the grey matter volume of overlap part. c The overlap of the cluster and Left Insula, and the partial residual plot between PSS score and the grey matter volume of overlap part. d The overlap of the cluster and Left amygdala, and the partial residual plot between PSS score and the grey matter volume of overlap part

With the whole-brain-based VBM analysis, we examined the effect of the interaction of age groups on the PSS. A cluster centered at the insula, extending to the OFC, and the left amygdala showed a significant interaction effect of stress by age on GMV, *t*(52) > 3.28, *p*_*corrected*_ < 0.001 (Fig. 1a, Tables 2 and 3) using whole-brain-based analysis. Non-parametric tests also showed that this cluster had a corrected *p* value less than .05.

**Table 2:** Interaction of stress by age on GMV

Anatomical regions	Cluster size (# voxels)	MNI Coordinates(mm)			T-Score	Corrected p
		X	Y	Z		
Frontal orbital cortex	1423	-32	6	−15	3.28	0.002
Left insular						
Left amygdala

**Table 3:** Correlation between PSS score, cortisol, and GMV of regions

Group			Cortisol	VBM clusters	OFC	Insula	Amygdala
Adolescent	PSS	r	0.01	0.68**	0.60**	0.60**	0.66**
		p	0.97	<0.01	<0.01	<0.01	<0.01
Adult	PSS	r	−0.52**	−0.43*	−0.49*	−0.42*	−0.26
		p	<0.01	0.03	0.01	0.03	0.19

Gender and IQ were added as covariates in the analysis

Average GMV: the average GMV of the cluster showing a significant interactive effect of stress by age

*indicates a significant statistical difference with *p* < 0.05

**indicated a significant statistical difference with *p* ≤ 0.01

The average volume of the cluster showing a significant interactive effect of stress by age was extracted from each individual and submitted to a set of post-hoc analyses. For each age group, a regression analysis was conducted between the average GMV and the PSS score, controlling for gender and IQ. A significant positive correlation was found between average GMV and the PSS score (r^2^ = 0.54, *p* < .01) in the group of adolescents, and a significant negative correlation was found between average GMV and the PSS score in adults (r^2^ = 0.14, *p* = .04). Furthermore, the same pattern was observed when we examined the three regions (insula, OFC and amygdala) in the cluster individually (Fig. 1b–d).

### Plasma cortisol and stress

**Fig. 2:**
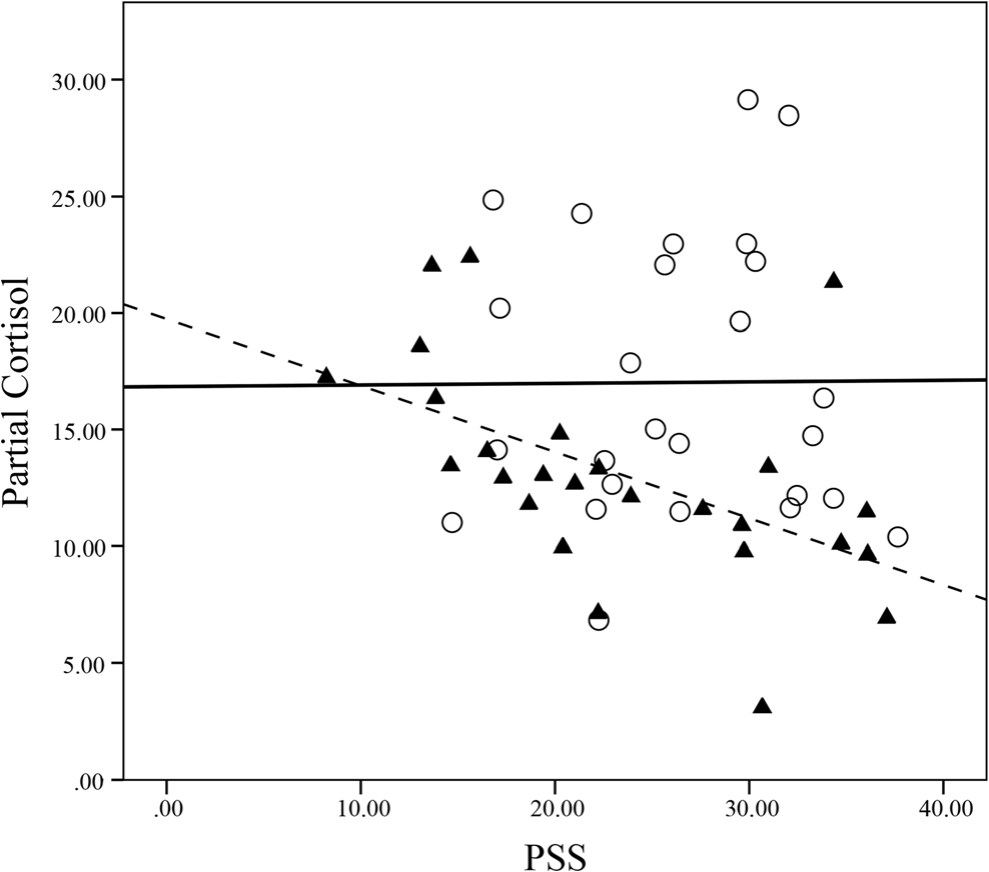
Partial residual on cortisol versus PSS score. Only the component predicted by the PSS score plus the residual were kept

To examine whether the cortisol level is related to the PSS score, correlation analysis was performed between the cortisol level and PSS score using SPSS 24.0 (IBM Corporation, Armonk, NY). In adults, the negative correlation between the cortisol level and the PSS score was significant (*p* < .05), whereas in adolescents, the relationship between the two variables was not significant (*p* > .05) (Fig. 2 and Table 3).

## Discussion

The findings of this study supported our a priori hypotheses that differences existed in the relationships between perceived stress and the structural volume of the OFC extending to the insula and amygdala. Specifically, the volume of this cluster was positively correlated with the PSS score in adolescence, whereas it was negatively correlated with the PSS score in adults. Also, a negative correlation was found between plasma cortisol and perceived stress, the volume of the OFC, or the insula in adults but not in adolescents.

### Brain structure and perceived stress

In terms of neuroimaging findings, the cluster showing stress-by-age interaction in volume covered the OFC, insula, and amygdala, which are closely related to stress-related perceptions, processes, and regulations. In particular, the results indicated that the grey matter volumes of these regions are positively correlated with the PSS in an adolescent while being negatively correlated in adults. Previous studies showed that the OFC plays an important role in processing emotion and social regulation (e.g. Rolls 2017) to adapt to the changing environmental contingencies. The findings of functional neuroimaging studies showed that the OFC supports social-emotional behaviors (Bachevalier and Loveland 2006). When the OFC is damaged, stress-related diseases become increasingly prevalent (Jackowski et al. 2012; Rolls 2017). The insula is known to be responsible to the processes of interoception, awareness of emotion, and stress responses (Craig 2009b; Gu et al. 2013). The activities and integrity of the insula have been found to be related to many stress-related diseases, such as major depression disorder (Avery et al. 2014; Zou et al. 2018), anxiety disorders (Alvarez et al. 2015; Baur et al. 2013), and PTSD (Klabunde et al. 2017; Zhang et al. 2016). The amygdala is one of the key neural correlates of affective processing, especially when challenged by stressful events (McEwen et al. 2016; Yamamoto et al. 2017).

Evidence showed that these regions continue to develop until adulthood (Gogtay et al. 2004; Pattwell et al. 2016), and during development, stress plays an important role in shaping these regions’ structures and functions (McEwen et al. 2016). We rationalize the findings by proposing that during the process of stress perception and regulation, the PFC plays an important role in top-down regulation (Cunningham et al. 2002) to the limbic regions. As the PFC is not yet mature in adolescence (Gogtay et al. 2004), the effect of the PFC’s top-down control to the limbic system may be weak. Therefore, the amygdala and insula received more stress stimulations, which led to more activation and bigger volumes. As feedback, the volume of the PFC, especially the OFC, also tends to increase. On the other hand, the top-down regulation model and the PFC in adults are fully developed. The amygdala and insula receive top-down control from the PFC during stress processing, which may limit their activities and volumes. In addition, the neurotoxic effect of stress could lead to decreased volumes in these regions (Lupien et al. 2018).

Animal models previously illustrated that stress could cause structural alteration of the brain, for instance, reduction of astrocyte density in the hippocampus (Ritchie et al. 2004) and disrupted dendritic spine plasticity in neurons of the perirhinal cortex (Gong et al. 2018) and decrease the number of GFAP^+^ astrocytes within the prefrontal cortex (Tynan et al. 2013). These physical consequences of enduring stress are suggested to be related to cognitive function loss. Unexpectedly, the results of this study showed no significant correlation between perceived stress and the volumes of the hippocampus, vmPFC, and ACC. Although the response to stress between adults and adolescents might be different, these regions closely related to stress do not seem to be the mechanism behind the age difference.

### Cortisol and perceived stress

In terms of cortisol-level findings, the results showed that the plasma cortisol level has a negative correlation with perceived stress in adults only. As for adolescents, no correlation between the cortisol level and perceived stress was indicated. This is consistent with previous findings. Milam et al. (2014) explored the relation between perceived stress and hair cortisol in healthy adolescents, with the result showing no significant associations between perceived stress and hair cortisol. Also, the findings of De Vriendt et al.’s study (2011) did not reveal stable relationships between perceived stress measured by an adolescent stress questionnaire and wake-up salivary cortisol in adolescents. Combing the results from the current and previous studies, we suspect that the relationship between the cortisol level and perceived stress might not be stable in adolescents due to developmental factors. Studies showed that both the basal HPA axis activity and cortisol reactivity are not fully matured and fluctuate throughout childhood and adolescence (Gunnar and Donzella 2002). During adolescence, experience plays a role in shaping the basal rhythms and reactivity of the HPA system (Gunnar and Quevedo 2008). Studies showed that long-term stress and trauma could cause the down-regulation of the HPA axis, which presents as hypoactive functioning (Hinkelmann et al. 2013). The previous literature suggested that the constant hyperactive functioning of the HPA axis due to long-term stress exposure could ultimately lead to the exhaustion of the system. This phenomenon of HPA axis overload has been reported in healthy adults (Faresjö et al. 2013) and patients with stress-related diseases, such as post-traumatic disorder (Meewisse et al. 2007), major depressive disorder (Bremmer et al. 2007), chronic fatigue syndrome (Gur et al. 2004), and burnout (Pruessner et al. 1999).

### Limitations

Results from this cross-sectional study should be interpreted with the following considerations. Although the results showed the relationship between perceived stress measures and brain structures in adolescents and adults, readers should not assume that the developmental trajectory of these relationships between the two age groups is linear. We selected early-morning cortisol levels as an indicator of observation. Although it is a common indicator of a person’s cortisol level among research, it should not be deemed as reflecting the baseline of a person’s cortisol level for the day, due to alterations throughout the day from the circadian rhythm. The abovementioned limitations could be addressed with future studies using a longitudinal design and cortisol extraction at multiple time points throughout a day. Addressing these issues could lead to a better understanding of the developmental trajectories of perceived stress and the brain structure, as well as the validity of using the morning cortisol level as a stress indicator.

## Conclusions

In sum, our findings suggested that the relationships between perceived stress and the structure of the brain appear to be age dependent and are different between adolescents and middle-aged adults. Perceived stress, an indicator of a stress-related impact on the brain, is more sensitive than cortisol measurement is in adolescents. We speculated that the developmental neural plasticity of the OFC, insula, and amygdala may be key neural correlates responsive to perceived stress in the adolescent brain. The findings of this study shed light on the possible vulnerability of the adolescent brain to stress as these adolescents perceived it. Future longitudinal studies are required for determining the developmental trajectories of these relationships.
